# An analysis of Cyclin D1, Cytokeratin 5/6 and Cytokeratin 8/18 expression in breast papillomas and papillary carcinomas

**DOI:** 10.1186/1746-1596-8-8

**Published:** 2013-01-18

**Authors:** Yu Wang, Jin-fu Zhu, Ying-ying Liu, Gui-ping Han

**Affiliations:** 1 Department of Pathology, The 2nd affiliated hospital of Harbin Medical University, Xuefu Road 246, Harbin, Nanggang District, China

**Keywords:** Cyclin D1, Cytokeratin, Papillary carcinoma, Papilloma, Double immunostaining

## Abstract

**Background:**

To evaluate the expression levels of Cyclin D1 in breast papillomas and papillary carcinomas, and to analyze the types of cells that co-express Cyclin D1 with Cytokeratin 5/6 (CK 5/6) or with Cytokeratin 8/18(CK 8/18).

**Methods:**

Fifty-nine cases of papillary lesions including 36 papillomas and 23 intracystic papillary carcinomas were examined. Cyclin D1, CK 5/6 and CK 8/18 expression levels were evaluated by double immunostaining.

**Results:**

Cyclin D1 is highly expressed in papillary carcinomas (27.54% ± 15.43%) compared with papillomas (8.81% ± 8.41%, p < 0.01). Cyclin D1 is predominantly expressed in Cytokeratin 8/18- expressing cells, rather than in Cytokeratin 5/6-expressing cells, regardless of the type of lesion. In Papillomas, Cyclin D1 exhibited a mean 11.42% (11.42% ± 10.17%) co-expression rate with Cytokeratin 8/18 compared with a mean 2.50% (2.50% ± 3.24%) co-expression rate with Cytokeratin 5/6 (p < 0.01). In papillary carcinomas, Cyclin D1 exhibited a mean 34.74% (34.74% ± 16.32%) co-expression rate with Cytokeratin 8/18 compared with a co-expression rate of 0.70% (0.70% ± 0.93%) with Cytokeratin 5/6 (p < 0.01).

**Conclusions:**

The increase in Cyclin D1 suggests an association of Cyclin D1 staining with papillary carcinomas. Although Cyclin D1 is an effective marker for the differential diagnosis of other papillary lesions, it cannot be used to distinguish between papilloma and papillary carcinoma lesions because its expression occurs in both lesions. Our results show that Cyclin D1 and CK 5/6 staining could be used in concert to distinguish between the diagnosis of papilloma (Cyclin D1 < 4.20%, CK 5/6 positive) or papillary carcinoma (Cyclin D1 > 37.00%, CK 5/6 negative). In addition, our data suggest that Cyclin D1 is expressed only in the cancer stem or progenitor cells that co-immunostained with CK 8/18 in papillary carcinomas, and predominantly with CK 8/18 in the papillomas.

**Virtual slides:**

The virtual slide(s) for this article can be found here: http://www.diagnosticpathology.diagnomx.eu/vs/7299340558756848

## Background

Papillary breast tumors consist of proliferative mammary epithelial cells that invade the ductal lumen and form fibro-vascular stalks that may evolve into branching arborescent structures. Intraductal papillomas and papillary ductal carcinomas in situ (DCIS) are examples of papillary breast lesions. Despite the well-described histological features of these two tumors, it is occasionally difficult to distinguish between them because of overlapping microscopic characteristics [1]. The key histological feature used to delineate benign papilloma from papillary DCIS is the presence of myoepithelial cells, which is preserved in the former and scant or absent in the latter. CK 5/6, SMA (smooth muscle actin) and p63 are generally accepted markers for the differential diagnosis of papillomas and papillary carcinomas [2].

In recent years, the elevated expression of Cyclin D1 in papillary carcinomas compared with papillomas has attracted significant attention. Several studies have reported that Cyclin D1 expression levels are sufficiently sensitive to distinguish between these two types of lesions [3]. Whether these two types of lesions can be distinguished by Cyclin D1 expression levels alone requires further investigation. Moreover, the mechanism underlying the elevated expression of Cyclin D1 in breast carcinomas remains unknown.

Cyclin D1 is a cell -cycle regulator that is essential for progression through G1 phase and is a candidate proto-oncogene. This protein has also been implicated in the pathogenesis of several human tumor types including breast carcinomas. To the best of our knowledge, there are no available published studies regarding the expression of Cyclin D1 in different breast cell types. Moreover, the use of Cyclin D1 alone to make a differential diagnosis between papilloma and papillary carcinoma remains a controversial topic. In this study, we aimed to evaluate the expression of Cyclin D1 in different cell types and to assess its potential to distinguish between papillomas and papillary carcinomas using a double immunostaining technique. Cells expressing CK 5/6 or CK 8/18 exhibited pale red or red-stained cytoplasm, whereas cells expressing Cyclin D1 exhibited a black nuclear staining pattern.

## Methods

We examined 59 papillary breast lesions (Table 1) from the database of the Department of Pathology of the 2nd affilliated hospital of Harbin Medical University, including 36 cases of intraductal papillomas and 23 cases of intracystic papillary carcinomas. All of the diagnoses were made with Cytokeratin 5/6, SMA, p63, CD10 and calponin.

**Table 1:** The distribution of the different age groups of patients with papillary lesions

**Age (year)**	**Papilloma**	**Papillary carcinoma**
16-39(mean 29.5)	11	2
40-59(mean 45.5)	19	16
60-76(mean 67.4)	6	5

Double immunostaining analyses were performed. The tissues were sectioned into sequential slices (two serial sections were cut from each block, specifically, one for Cyclin D1 and CK 5/6 staining, and one for Cyclin D1 and CK 8/18 staining). Initial immunostaining was performed using a Cyclin D1 antibody purchased from QuanHui (Beijing, China), and a Cytokeratin 5/6 (CK 5/6) or Cytokeratin 8/18 (CK 8/18) antibody, both of which were purchased from Maxim Bio (Fuzhou, Fujian, China). Each primary antibody was applied at a final dilution of 1:50. The Maxim DouMax Vision^tm^ kit was purchased from Maxim Bio, which included Endogenous Peroxidase Blocking Solution, Non-Immune Serum, Biotinylated Secondary Antibody, Streptavidin Alkaline Phosphatase, BCIP/NBT, Amplifer, Streptavidin Peroxidase, AEC Buffer, AEC Substrate, AEC Chromogen, Hematoxylin, and ClearMount.

All tissues were fixed in a 10% formalin solution. Four micron-thick serial sections were generated from paraffin-embedded blocks. For epitope retrieval, the slides were boiled in a cooker in EDTA buffer pH 9.0 for 20 minutes. Next, the sections were incubated with the Cyclin D1 primary antibody over-night at 4 degrees centigrade. The remaining steps of the procedure were performed according to the manufacturer’s recommended protocol. The sections were visualized by incubation in BCIP/NBT for 30 minutes at room temperature. The nuclei of cells that expressed Cyclin D1 were stained black.

Next, the sections were treated with Amplifer for 10 minutes, incubated in 10% normal goat serum for 30 minutes at room temperature, and incubated with a second primary antibody to either CK 5/6 or CK 8/18 for 1 hour at room temperature. The remaining steps of the procedures were performed according to the manufacturer’s recommended protocol. The sections were visualized using a cocktail of AEC Buffer, AEC Substrate and AEC Chromogen for 10 minutes at room temperature. The cytoplasm of CK 5/6 or CK 8/18-expressing cells appeared pale red or red after staining. Hematoxylin was used as a counter stain, and the nuclei were thus stained blue-purple, unless the nuclei were positive for Cyclin D1: in those cases, the nuclei were stained black.

Next, Cyclin D1, CK 5/6 or CK 8/18 staining was classified as either negative or positive for each antibody. Five fields were selected randomly at magnification of 400×. The number of Cyclin D1 expressing cells and, CK 5/6 or CK 8/18 co-expressing cells was quantified – out of 200 cells for every selected field. The mean expression rate of Cyclin D1 was calculated. Data were analyzed using the SPSS 13.0 software package (IBM, Armonk, NY, USA) and presented as $\bar{X}$±SD. The Mann–Whitney U test was used to analyze discrepancies. *P*-values < 0.01 were considered to be statistically significant.

## Results

**Figure 1:**
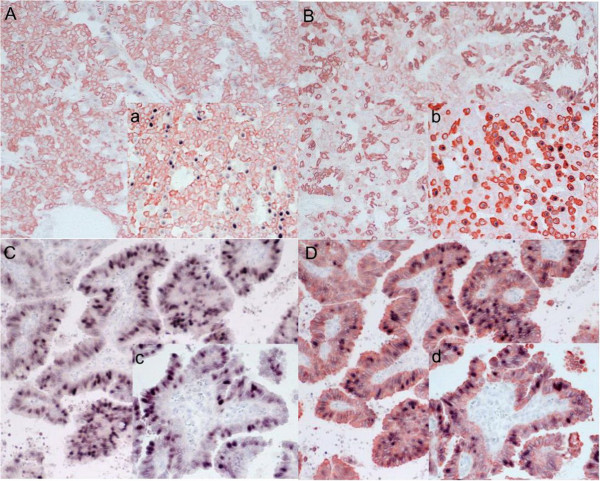
**Immunohistochemical double staining for Cyclin D1 and CK 5/6 or CK 8/18 in papilloma (A B, original magnification × 200; a b, original magnification × 400) and in papillary carcinoma (C D, original magnification × 200; c d, original magnification × 400). **The cytoplasm of the cells expressing CK 5/6 is stained pale red and CK 8/18 was stained red, whereas the cytoplasm of cells expressing CK 8/18 is stained red. The nuclei of Cyclin D1 positive cells are stained black. (**A**, **a**) Immunohistochemical double staining for Cyclin D1 and CK 5/6 in a papilloma. The Cyclin D1 positive cells were hardly detected in (**A**). (**B**, **b**) Immunohistochemical double staining for Cyclin D1 and CK 8/18 in a papilloma. There were a few Cyclin D1 positive cells detected in (**B**). Upon comparing (**a**) and (**b**), Cyclin D1 expression is clear in the cells that are positive for CK 8/18 in contrast to the cells that are positive for CK 5/6 in the papillomas. (**C**, **c**) Immunohistochemical double staining for Cyclin D1 and CK 5/6 in papillary carcinoma. The cells are not immunoreactive for CK 5/6. (**D**,**d**) Immunohistochemical double staining for Cyclin D1 and CK 8/18 in papillary carcinomas. Cyclin D1 (**D**) localizes in practically the same position compared with (**C**). The number of the Cyclin D1 positive cells (**C**, **D**) clearly increases, in contrast to (**A**) and (**B**). As in the papillomas, Cyclin D1 only appears in the cells that co-express CK 8/18.

**Figure 2:**
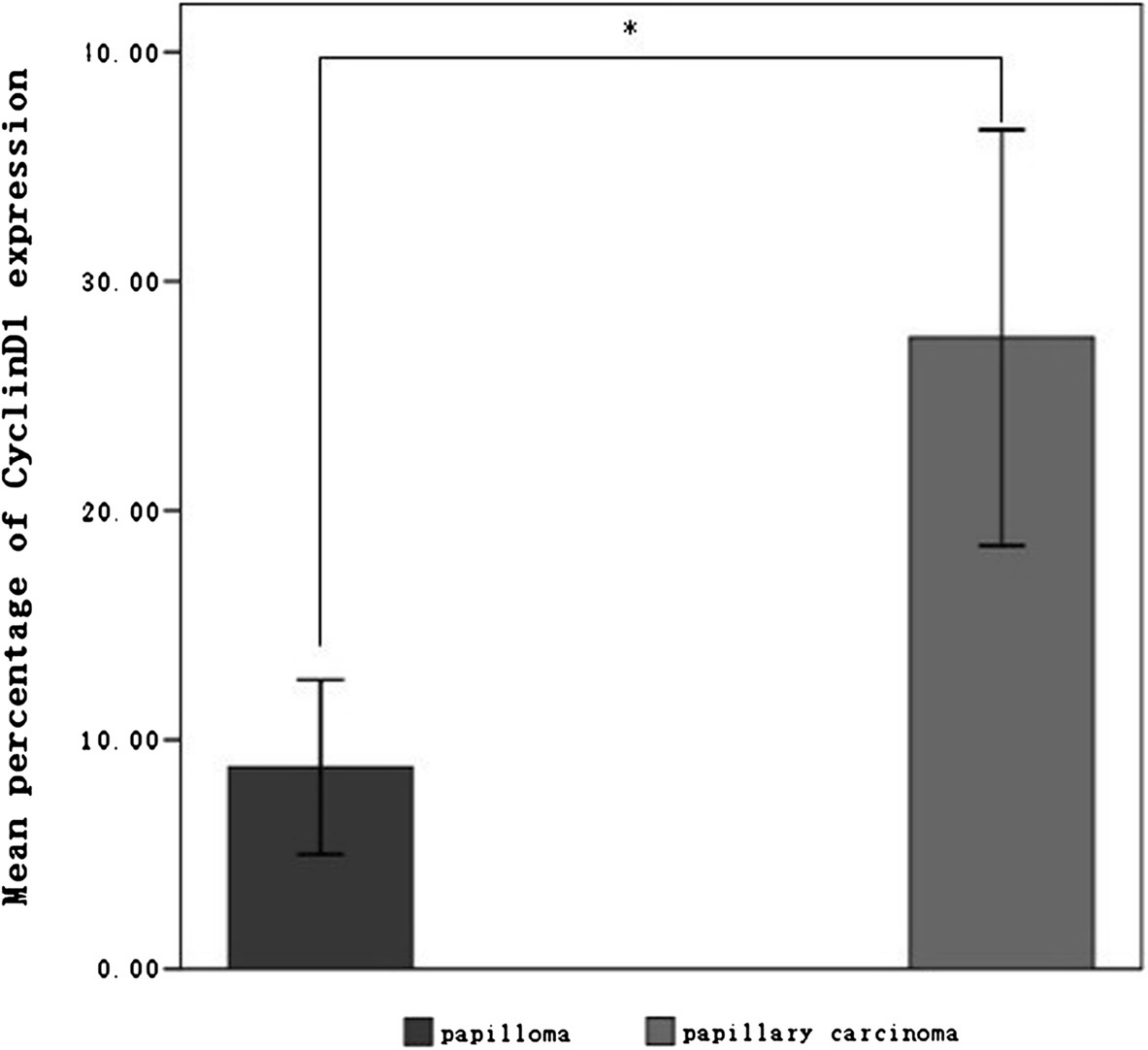
**The mean expression rate of Cyclin D1 in the papillomas and the papillary carcinomas.** The expression rate of Cyclin D1 is 27.54% ± 15.43% in papillary carcinomas and 8.81% ± 8.41% in papillomas (p < 0.01). The values are expressed as $\bar{X}$±SD. *P < 0.01 (Mann–Whitney U test). The difference exhibited clear statistical significance.

We detected negligible levels of Cyclin D1 expression within papilloma sections (shown in Figure 1A, B) compared with high levels of Cyclin D1 expression in papillary carcinomas (shown in Figure 1C, D). In keeping with these results, the mean rate of Cyclin D1 expression in the papillomas was 8.81% (8.81% ± 8.41%), whereas the mean expression rate of Cyclin D1 in the papillary carcinomas exhibited a statistically significant increase to 27.54% (27.54% ± 15.43%, p < 0.01, Figure 2).

In our study, the rate of Cyclin D1 expression in papilloma cells was 0-37%, whereas the rate of Cyclin D1 expression in papillary carcinoma cells was 4.20%-60.80%. Although there was overlap in Cyclin D1 expression between these two lesions, a small number of papilloma cases exhibited higher Cyclin D1 expression than the average Cyclin D1 expression of the papillary carcinomas and vice versa, the Cyclin D1 together with CK 5/6 staining could be used to distinguish between papillary carcinoma and papilloma samples. Statistical analysis indicated that Cyclin D1 expression could distinguish papilloma from papillary carcinoma (p < 0.01). If the percentage of the Cyclin D1 expression was over 37.00% and negative for CK 5/6 expression, a diagnosis of papillary cacinoma was made. Conversely, if the percentage of the Cyclin D1 expression was below 4.20% and posirive for CK 5/6 expression, a diagnosis of papilloma was made.

**Figure 3:**
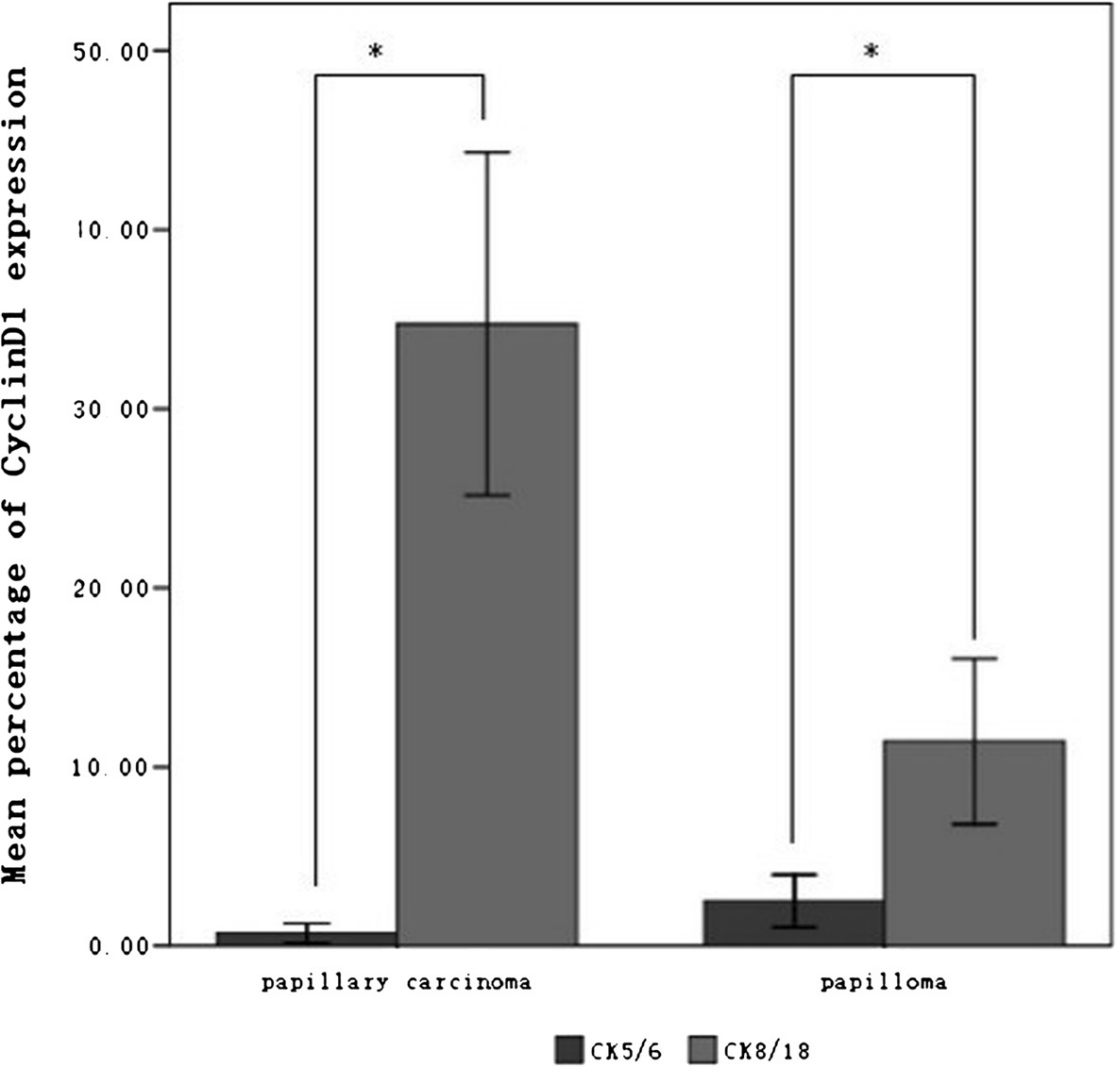
**The mean co-expression rate of Cyclin D1with CK 5/6 or CK 8/18 in papilloma and papillary carcinoma cells. **The mean co-expression rate of Cyclin D1 with CK 8/18 was 11.42% ± 10.17%, and that with CK 5/6 was 2.50% ± 3.24% (p < 0.01); whereas in papillary carcinomas, the mean co-expression rate of Cyclin D1 with CK 8/18 was 34.74% ± 16.32%, and that with CK 5/6 was 0.70% ± 0.93% (p < 0.01). The values are expressed as $\bar{X}$±SD.*P < 0.01 (Mann–Whitney U test). The differences exhibited statistical significance.

In the papillomas, Cyclin D1 was predominantly expressed in CK 8/18 positive cells with limited expression CK 5/6 positive cells (shown in Figure 1a, b). The mean co-expression rate of Cyclin D1 with CK 8/18 was 11.42% (11.42% ± 10.17%), whereas the mean co-expression rate of Cyclin D1 with CK 5/6 was 2.50% (2.50% ± 3.24%, p < 0.01, Figure 3). Thus, there is a statistically significant difference in the co-expression rates of Cyclin D1 and CK 8/18 or CK 5/6 between these two lesion types. Cyclin D1 was almost exclusively expressed in the cells that co-expressed CK 8/18, whereas CK 5/6 is almost never expressed in the papillary carcinomas (Figure 1c, d). The mean co-expression rate of Cyclin D1 with CK 8/18 is 34.74%(34.74% ± 16.32%), and the mean co-expression rate of Cyclin D1 with CK 5/6 is 0.70% (0.70% ± 0.93%, p < 0.01, Figure 3), indicating that the overexpression of Cyclin D1 is significantly correlated with cells expressing CK 8/18.

## Discussion

Stem cells found within the breast tissue have the ability to self-renew and generate daughter cells, including all of the cell types found in mature breast tissues [4]. Multi-potent mammary stem cells (MaSCs) produce committed progenitors, which subsequently differentiate into myoepithelial and luminal epithelial cells [4]. During the differentiation process, the self-renewal capacity of the cells is gradually lost. Of the MaSCs and the committed progenitors, it is believed that at least one is a bi-potent progenitor. These progenitors, which include bi-potent progenitors, all express basal markers, such as CK5, CK6, and CK14 [5]. In addition to those progenitors previously mentioned, the luminal progenitors also express such markers as CK8 and CK18. Consequently, the existence of a luminal progenitor population has been identified [6], but the myoepithelial progenitor could not be identified because the progenitors also express basal markers, similar to MaSCs [4]. Myoepithelial progenitors differentiate into the myoepithelial cells, and luminal progenitors differentiate into cells that are restricted to either ductal or alveolar lineages. The terminal luminal epithelial cells or alveolar cells lose the basal markers, expressing only CK8 and CK18, whereas the terminal myoepithelial cells maintain the expression of basal markers CK5, CK6, CK14 and the new myoepithelial marker P63, SMA (smooth muscle actin), and calponin.

These findings form the basis of the cancer stem cell (CSC) hypothesis, which posits that certain tumors are initiated by one or more self-renewing CSCs that differentiate into large populations of non-self-renewing but rapidly dividing, cells which are responsible for generating the tumor mass [7]. The cancer stem cells could potentially be derived from bi-potent stem cells or from more differentiated cells that have acquired self-renewal capabilities. In contrast to the normal stem cells, the cancer stem cells lose their capacity for multi-directional differentiation and only produce tumors with features of a particular lineage, including luminal or basal like lineages. Regardings to tumor heterogeneity, there still remains controversy. Current explanations comprise two predominant models: the cancer stem cell hypothesis and the clonal evolution and selection hypothesis. According to the clonal evolution hypothetical model, tumor cell phenotypes are determined by the phenotype of the original cell type of the tumor-initiating cell. In this study, papillary carcinoma cells expressing CK 8/18 should be differentiated from one cancer stem cell/progenitor of luminal cell lineage, and accordingly, the luminal progenitor or terminal cells should express the same markers. This phenomenon would explain the clonal evolution of papillary carcinomas. In our study, the papilloma masses consisted of cells that were positive for CK 5/6 and CK 8/18, clearly indicating that these cells originated from different tumor stem/progenitor cells. Taken together, the two models indicate that the cellular phenotypes are unstable and can change as the tumor evolves. The heterogeneity of the papilloma cells can be considered to characterize a stage of the tumor progression, specifically, a competition among tumor cells with different phenotypes. It is possible that certain papillary carcinomas derived from the papillomas might result from the CK 8/18-positive tumor cells that replace the CK 5/6 positive cells. The two models are complementary in explaining tumor heterogeneity and progression. However, the hypothesis that the papillary carcinoma cells could be derived from the papilloma cells which have the same progenitor of the cancer cells, requires further research. However, the validity of the explanation above regarding tumor progression remains to be confirmed.

In our results, Cyclin D1 staining was predominantly detected in the cells that were also immunoreactive for CK 8/18 in either the papillary carcinomas or papillomas. Previous studies have demonstrated a correlation between Cyclin D1 over-expression and breast cancers. The hypothesis that papillary carcinoma cells could be derived from papilloma cells which have the same progenitors as the cancer cells might be explained by the expression pattern of Cyclin D1 in cells co-expressing CK 8/18.

Cyclin D1, one of the protein mediators of the G1/ S cell-cycle transition, is commonly altered in breast cancer and contributes to tumorigenesis, presumably by increasing proliferation [8]. It is generally accepted that the initiation of most tumors is triggered by chromosomal instability (CIN), which is characterized by chromosomal abnormalities and an altered gene expression profile. Data from Mathew et al. [9] suggest that Cyclin D1 contributes to CIN and tumorigenesis by directly regulating a transcriptional program that governs chromosomal stability. During maturation of the breast cell, the differentiation and proliferative behaviors are regulated by a series of signaling pathways, such as the Notch [10], Hedgehog [11], and Wnt [12] pathways. The CCND1 gene encodes a subunit of the Cyclin D1 holoenzyme, which can phosphorylate and inactivate pRB and NRF1 to regulate nuclear synthesis and mitochondrial biogenesis [13-17]. The biological effects of Cyclin D1 overexpression in the process of tumorigenesis are exhibited through the pathways mentioned above. Several studies have demonstrated that the disrupted regulation of these pathways can lead to the development of breast cancer in mice [18-21] and in humans [22-24]. One report indicated that deletion of the CCND1 gene leads to failed mammary gland development in mice [25]. Another study demonstrated that overexpression of the Cyclin D1 oncogene can induce mammary gland tumors in mice [26]. These findings further suggest that Cyclin D1 might directly or indirectly trigger the differentiation of mammary glandular cells. Other studies have suggested that Cyclin D1 might inhibit the differentiation of adipocytes [8]. In addition, many studies have indicated that high Cyclin D1 expression levels correlate with CIN, specifically in the luminal B subtype tumors [9]. This phenomenon provides a possible explanation for why Cyclin D1 was mainly expressed in the cells that expressed CK 8/18 but not in those that expressed CK 5/6. Cyclin D1 over-expression leads to the sustained proliferation of mammary epithelial cells, which is associated with a delay in acinar development in vitro models [27] and a failure to undergo terminal differention in mouse models [28]. The cells in either the papillomas or the papillary carcinomas that expressed CK 8/18 could be derived from the same cancer stem/progenitor cells, which exhibit the capacity of self-renewal and strict luminal differentiation, and which over-express Cyclin D1 proteins because of various genetic or epigenetic events. The distinct expression patterns of Cyclin D1 between the papillomas and papillary carcinomas might be explained by their occurrence during different stages of tumorigenesis.

## Conclusions

In this study, we evaluated the levels of Cyclin D1 expression in CK 5/6- or CK 8/18-positive cells from breast papilloma and papillary carcinoma. Double immunostaining was used to analyze the types of cells that co-express Cyclin D1 with CK 5/6 or CK 8/18. Increased levels of Cyclin D1 are associated with inreased likehood of papillary carcinomas. This study also demonstrated the usefullness of CK 5/6 in distinguishing breast papilloma (Cyclin D1 < 4.20%) from papillary carcinoma (Cyclin D1 > 37.00%). During the differentiation of breast cells, various immunochemical markers appeared or disappeared at different time-points. In our results, Cyclin D1 was almost exclusively expressed in the tumor cells stained by CK 8/18, which is a marker of luminal cells in normal breast during development into the final luminal format. We therefore conclude that Cyclin D1 is expressed exclusively in the cancer stem or progenitor cells that positively co-immunostained for CK 8/18 in papillary carcinomas and predominantly for CK 8/18 the papilloma lesions.

## Abbreviations

CK 5/6: Cytokeratin 5/6; CK 8/18: Cytokeratin 8/18; DCIS: Ductal carcinomas in situ; MaSCs: Multipotent mammary stem cells; SMA: Smooth muscle actin; CSC: Cancer stem cell; CIN: Chromosomal instability.

The Project Fund: the Scientific Research Foundation for the Returned Overseas Chinese Scholars, State Education Ministry (2009–1001).

## Competing interests

The authors declare that they have no competing interests.

## Authors’ contributions

YW and JZ carried out the experiments and analysis of results obtained. All authors participated in the design of the study, analysis of obtained results. YW drafted the manuscript. All authors have read and approved the final manuscript.
